# Validation of the Jefferson health-related social needs screener

**DOI:** 10.3389/frhs.2025.1658661

**Published:** 2025-09-19

**Authors:** Sara Beachy, Kristin L. Rising, Richard W. Hass, Terry Hyslop, Isabella Muti, Mackenzie Kemp, Rhea E. Powell, Cara Martino, Baligh R. Yehia, Joseph G. Cacchione, Patricia Henwood

**Affiliations:** ^1^ Department of Psychiatry, Sidney Kimmel Medical College, Thomas Jefferson University, Philadelphia, PA, United States; ^2^ Center for Connected Care, Thomas Jefferson University, Philadelphia, PA, United States; ^3^ Department of Emergency Medicine, Sidney Kimmel Medical College, Thomas Jefferson University, Philadelphia PA, United States; ^4^ College of Population Health, Thomas Jefferson University, Philadelphia, PA, United States; ^5^ Department of Pharmacology, Physiology, and Cancer Biology, Thomas Jefferson University, Philadelphia, PA, United States; ^6^ Sidney Kimmel Medical College, Thomas Jefferson University, Philadelphia, PA, United States; ^7^ Department of Medicine, Sidney Kimmel Medical College, Thomas Jefferson University, Philadelphia, PA, United States; ^8^ Jefferson Health, Philadelphia, PA, United States

**Keywords:** health-related social needs, social determinants of health, health disparities, health equity, psychometrics

## Abstract

**Introduction:**

Screening for health-related social needs (HRSN) is a growing national health priority. While multiple HRSN screening tools currently exist, none to our knowledge have been evaluated using robust statistical analyses. The goal of this work is to provide results from a validation study of the Jefferson HRSN screener conducted across inpatient and outpatient settings.

**Methods:**

This retrospective cross-sectional psychometric study included HRSN assessments conducted across inpatient and outpatient settings with adult patients from March 2023 to May 2024. The study was conducted across a 17-hospital academic health system serving a diverse community in a 9-county area crossing two states**.** Participants answered the HRSN screener, which includes eight questions across seven HRSN domains (financial, food, housing, utilities, transportation, violence/safety, and social connection) and two follow up questions, as part of standard healthcare encounter procedures. The measure was assessed with item response theory and a two-parameter logistic model. A follow-up analysis using Latent Class Analysis (LCA) was used to assess whether HRSN items and demographic variables could be used to identify people with higher levels of social vulnerability index (SVI). Higher SVI indicates higher levels of needs based on community and neighborhood related factors.

**Results:**

The final sample included data from 302,929 adults. Patients were relatively evenly distributed across ages (< 45 years, 32%; 45–64 years, 32%; 65–84 years, 30%; 85+, 4%). Most patients were Non-Hispanic (87%), White (66%), and female (59%). A third of patients were in the medium-high (18%) and high (15%) SVI areas. Positive responses across questions ranged from 0.90%–5.90%. Slopes ranged between 1.67–3.77, and difficulty parameters ranged between 2.20–3.31, indicating that the items can detect a high level of need. LCA results suggested that the eight HRSN items combined with basic demographic variables could help identify people with higher HRSN.

**Discussion:**

The Jefferson HRSN screener provides a valid approach for HRSN screening across healthcare settings. The eight screening questions, combined with additional questions to evaluate the patient's desire for help and urgency, can be used to identify patients needing additional resources to address fundamental social needs potentially contributing to health disparities.

## Introduction

The national discourse on how social risk factors impact individuals' health and the importance of addressing these factors during healthcare encounters has been growing in recent years. Broadly, social risks include geographical location and a person's economic and social positioning in a community may negatively impact a person's functioning, quality of life, and mental and physical health. Multiple entities have sought to further define social risks to implement policies that may address these factors. Frequently used social risks frameworks include social determinants of health (SDOH) and health-related social needs (HRSN). In late 2023, the White House released The U.S. Playbook to Address Social Determinants of Health (1), and the Department of Health and Human Services (HHS) put out a complementary Call to Action entitled “Addressing Health-related Social Needs in Communities Across the Nation” (2). HHS defines SDOH as “the conditions in the environments where people are born, lives, learn, work, play, worship, and age that affect a wide range of health, functioning, and quality-of-life outcomes and risks” (3), and HRSN as the “social and economic needs that individuals experience that affect their ability to maintain their health and well-being” (2). SDOH and HRSN are closely related concepts that both affect health: SDOH are community-level factors, such as availability of nutritious foods and affordable housing in a community, while HRSNs are individual-level factors (2). HRSNs include factors such as financial strain, food insecurity, transportation barriers, housing instability, and social isolation. Unaddressed HRSN affect individuals' ability to maintain their health and well-being and can be significant contributors to poor health outcomes.

Routine HRSN screening in the health care setting may help healthcare systems and clinicians improve health outcomes by identifying and addressing unmet HRSN, thus increasing the value of care provided and decreasing healthcare disparities. While routine screening for some HRSN has increased in recent years, prior studies have reported varying estimates for comprehensive HRSN screening across hospitals in the United States (4, 5). Further, amongst hospitals and healthcare facilities evaluated that perform HRSN screening, many were not using screening tools that comprehensively address the 5 key domains of social needs: food security, housing stability, utility access, transportation access, and interpersonal violence (4). Recognizing the importance of HRSN screening in health systems, the Centers for Medicare and Medicaid Services (CMS) added two quality measures for inpatient stays in the fiscal year 2023 Hospital Inpatient Prospective Payment Rule (6), requiring reporting of screening for social drivers of health by January 2024. CMS also included opportunities for billing for social needs screening and for community health workers addressing HRSN in the outpatient physician fee schedule starting in 2024, though clarified the requirement for a validated tool in order to charge for these services (7).

Beyond CMS requirements to screen for HRSN, other empirical literature suggests that HRSN can be used to predict healthcare utilization and health outcomes. Broadly, risk models have had difficulty in accurately predicting outcomes; yet, the inclusion of HRSN has been found to improve the predictive abilities of these models (8). Additionally, the use of HRSN can be used clinically to triage vulnerable patients to appropriate services, particularly for individuals from disadvantaged neighborhoods who are at increased risk for social determinants of health and subsequent adverse health outcomes (9). However, psychometric data for HRSN screeners remains variable and limited. Indeed, two systematic reviews of HRSN measures found that measure quality across all identified measures was low with few studies that adequately assessed validity (10–12). Of the measures that were reviewed, none to our knowledge evaluated the measures using robust statistical methods, such as item response theory (IRT) or classical test theory, or follow up analyses to assess validity.

IRT and latent class analysis (LCA) are both advanced statistical procedures that have been successfully employed in measure development and validity studies. IRT is a gold standard method for evaluating psychometric properties of measures. In IRT, items are hypothesized to be unidimensional and represent the amount of the latent trait that a person exhibits and the probability that a person will endorse this construct based on their experience of that latent trait. Furthermore, IRT assumes invariance, meaning it can measure the same construct across a heterogeneous sample as participant characteristics are independent from item parameters (13). Thus, IRT can help evaluate the construct validity of a measure across care settings and populations. LCA has been employed previously in psychology literature to assess the validity of diagnostic measures. In these studies, LCA was used to identify latent clusters of groups who are differentiated based on their responses to items (14–16). Allen and colleagues specifically used LCA after employing IRT to assess the validity of their newly developed measure, hypothesizing that identification of a 2-class solution indicates validity evidence (15). Legleye and colleagues used LCA to assess the validity of the screening abilities of a substance use measure (16). In this analysis, they used findings from LCA to ascertain whether the measure could be used to identify substance use problem severity.

In the following, we present results from a validation study using IRT and LCA of the Jefferson HRSN screener and provide the final validated screener for use across other health systems. Additionally, we demonstrate the utility of using the Jefferson HRSN screener to help identify groups who may be more at-risk.

## Materials and methods

### Design and setting

This is a retrospective cross-sectional study conducted at Jefferson Health and Thomas Jefferson University (TJU) in Philadelphia, PA. This study was conducted with data from 13 acute care hospitals and ambulatory care sites within the Jefferson Health system that was collected between March 2023 and May 2024 using the Epic Electronic Medical Record. Jefferson Health is a large comprehensive health system centered in Philadelphia, PA currently comprised of 32 hospitals that span two states and have catchment areas that cover more than 6.5 million residents. Jefferson's footprint within this market spans urban and suburban geographies and one of the most diverse socio-economic areas in the country. These areas have a wide range of income disparities, with an average poverty rate of 11.7% across the 15 counties and rates ranging from 21.7% in Philadelphia County to 5.3% in Chester County, PA (17). The Jefferson Health network provides a broad range of inpatient and outpatient services with more than 226,000 annual admissions, more than 870,000 annual emergency department visits and 8.8 million outpatient visits annually.

The Jefferson HRSN screener is comprised of 10 questions, including eight screening questions across seven HRSN domains (financial, food, housing, utilities, transportation, violence/safety, and social connection) and two follow-up questions. The two follow-up questions (“Do you want help?” and “Are your needs urgent?”) are only asked if at least one screening question is reported as positive. The screener was developed by our team to meet the needs across our large health system. Key considerations in screener development included having questions that were easy to both ask and answer, had a reading level of 6th grade or lower, had binary response options, and addressed issues that the health system could provide resource linkages and interventions. Leaders in the Jefferson Health system initially developed the questions based on existing screeners (18–23). (see Table 1). They ensured that they retained all domains that are typically included in HRSN measures based on the existing developed measures. They modified language of these existing screeners to simplify questions for readability and to ensure that each item that was included could be meaningfully addressed within a healthcare setting. The overall goal was to ensure ease of use and brevity so that the HRSN could be administered by a staff member and fit into clinical flow as opposed to solely relying on electronic dissemination of the screener which can lead to poorer response rates (24). Further, they wanted to ensure that the Jefferson Health system could be responsive to a positive screen using existing resources. Their goal was to leverage both community health workers and social workers who are embedded throughout the system to consult with patients who screened positive. These personnel have built extensive resource lists which would enable them to connect patients with community entities and organizations who can address patients' needs. The use of community health workers and social workers is typical of healthcare systems to address patient needs. Questions were further refined upon initial implementation across Jefferson health sites based on patient and clinician feedback, with the goal of having a survey tool that could be easily administered in both inpatient and outpatient healthcare settings and maintain patient care workflows while allowing for patients who desire help related to HRSN to be identified and linked to appropriate resources. For example, the safety question was modified to change the frame of reference from “where you currently live” to “in your home or where you currently stay”. This was done in response to the feedback that many individuals were interpreting “where you currently live” as their neighborhood as opposed to their home specifically, and the intention was to focus on individual-level (which are much more actionable) as opposed to neighborhood-level factors. (Table 1) Since initial refinement, the screener has not been further modified.

**Table 1:** Jefferson HRSN screener questions along with associated domains and as related to questions from other HRSN screeners.

Jefferson health HRSN screening questions	Other related HRSN screener questions	Domain
In the last 12 months did you skip medications to save money? Yes/No	**Arlington Screening Tool** (18) In the past year have you or any of your family members been unable to get any of the following when it was really needed. CHECK ALL THAT APPLY: Food, Clothing, Utilities Child care, Medicine or any health care (medical, dental, mental health or vision), Other (please write in notes), Do not have problems meeting my needs	Financial
	**Structural Vulnerability Assessment Tool** (19) Have you ever been unable to pay for medical care or for medicines at the pharmacy? Yes/No	
In the last 12 months, was there a time when you needed to see a doctor but could not because of cost? Yes/No	**Health Leads Social Needs Screening Toolkit** (20) In the last 12 months, have you needed to see a doctor, but could not because of cost? Yes/No	Financial
In the last 12 months did you ever eat less than you felt you should because there wasn't enough money for food? Yes/No	**Health Leads Social Needs Screening Toolkit**(20) In the last 12 months, did you ever eat less than you felt you should because there wasn’t enough money for food? Yes/No	Food
Are you worried that in the next 2 month you may not have stable housing? Yes/No	**Health Leads Social Needs Screening Toolkit** (20) Are you worried that in the next 2 months, you may not have stable housing? Yes/No	Housing
In the last 12months has the electric, gas, oil, or water company threatened to shut off services in your home? Yes/No	**American Academy of Family Physicians Social Needs Screening Tool**(21) In the past 12 months has the electric, gas, oil, or water company threatened to shut off services in your home? Yes/No/Already shut off	Utilities
In the last 12 months, have you ever had to go without healthcare because you didn't have a way to get there? Yes/No	**Health Leads Social Needs Screening Toolkit**(20) In the last 12 months, have you needed to see a doctor, but could not because of cost? Yes/No	Transportation
Are you concerned about your physical or emotional safety in your home or where you currently stay? Yes/No	**North Carolina Standardized SDOH Screening Questions** (22) Do you feel physically or emotionally unsafe where you currently live? Yes/ No	Violence/Safety
Do you often feel lonely? Yes/No	**Accountable Health Communities Screening Tool** (23) Supplemental: How often do you feel lonely or isolated from those around you? Never/Rarely/Sometimes/Often/Always	Social Connection
	**Health Leads Social Needs Screening Toolkit**(20) I often feel that I lack companionship. Yes/No	
Do you want help with any of your needs? Yes/No	**Health Leads Social Needs Screening Toolkit** (20) If you checked YES to any boxes above, would you like to receive assistance with any of these needs? Yes/No	
Are any of your needs urgent? Yes/No	**Health Leads Social Needs Screening Toolkit**(20) Are any of your needs urgent? For example: I don't have food tonight, I don't have a place to sleep tonight Yes/No	

Jefferson Health started routine use of the HRSN screener across primary care practices (approximately 100 practices) in March 2023 and across inpatient hospital units in September 2023. Outpatient screenings were conducted either by nursing or clinical staff asking the patients the questions and recording answers into the electronic health record (EHR) during the patient visit or through patients answering the questions directly into the EHR via a patient portal-generated questionnaire at appointment check-in or a paper questionnaire that was later transcribed into the EHR. The screening questions are asked once a year in the outpatient setting. For inpatient, the screener is done at every inpatient encounter by clinical personnel as part of the admission documentation in the EHR. All study activities were approved by the TJU institutional review board.

### Measures

Data for all patients who were over the age of 18 and were screened from March 2023 to May 2024 were included in this analysis. Data were only included for each patient once in analysis, using data from each patient's most recent HRSN screening. Visit level data that were extracted from the EHR included HRSN responses, age, sex at birth (male/female), race and ethnicity, and social vulnerability index (SVI).

#### Social vulnerability index

SVI is an index that was developed by the Centers for Disease Control and Prevention (CDC) as a tool to be used by community entities to understand the needs and develop adequate infrastructure to respond to these needs. (25) This index is comprised of four system-level domains which parallel the components of SDOH (i.e., socioeconomic status, household characteristics including disability, minority status, and housing and transportation) as well as the individual-level domains of the Jefferson HSRN screener. The index is integrated into Epic and is calculated for each patient based on their documented address. We applied this index as a measure of system-level SDOH to evaluate the validity of the Jefferson HSRN screener to capture the construct of SDOH at different levels (i.e., system and individual). SVI was coded as a categorical variable with four categories (Low = 0.00–0.24, Low Medium = 0.25–0.49, Medium High = 0.50 = 0.74, and High = 0.75–1.00), with Low SVI, indicating the least vulnerability, used as the reference category.

### Data analysis

Summary counts and percentages were calculated for each of the 10 questions in the HRSN screener and for all demographic variables. However, only the eight screening items were included in the IRT and LCA analyses. Multiple imputation was used to handle missing data. Prior to any analyses multiple imputation was conducted using Mplus v 8.9. Broadly, multiple imputation contains three processes: imputation, analysis, and pooling. Mplus uses Markov chain Monte Carlo (MCMC) simulation for multiple imputation and can include both categorical and continuous variables to generate a specified number of datasets (26). This method combines Markov chain with Monte Carlo procedures where random samples are drawn from a distribution using Markov chains which are then used to estimate probabilities in a target distribution that will be used for analyses. We used the variance covariance model which can include both continuous and categorical variables with categorical variables being converted to an underlying continuous variable. This model assumes all variables are dependent and produces an unrestricted variance-covariance matrix with categorical variables being defined as 1 on the diagonal (26). For the imputation process, we included all variables that would eventually be used in the analysis (i.e., eight HRSN items, race, ethnicity, age, SVI, encounter location, and whether patients had mychart). We generated 5 imputed datasets. We then conducted two analyses: IRT and latent class analysis (LCA), and Mplus v 8.9 was employed for all analyses.

#### Item response theory analysis

IRT was utilized to assess the initial validation of the 8-item measure. A fundamental assumption of IRT is that the measure is unidimensional and that items have local independence. IRT is used to determine how sensitive each item is to varying levels of a latent variable by providing information on the location (i.e., item difficulty) and the slope of each item in the measure (27). The location (*b*) identifies the point at which the probability that a person endorses a specific item is 50% while the slope (*a*) indicates the strength of the item in relation to the underlying construct. Larger location values indicate that individuals have higher amounts of the measured trait. Steeper slopes indicate stronger relationships with the underlying construct while less steep slopes indicate weaker relationships. Slopes typically range between 0.5 and 3.0 with slopes >4 indicating potential covariation (28). The unidimensional structure of the eight screening items was then assessed using the 5 imputed datasets using IRT and the two-parameter logistic model (2PL) was used for assessing discrimination. (29).

#### Latent class analysis

LCA employs probability-based analyses to group people based on their responses (30). Best practices for conducting an LCA require an iterative approach, comparing multiple solutions, and using multiple factors to identify the appropriate model. To identify the best model, we used a combination of the Akaike Information Criterion (AIC), the Sample-sized adjusted Bayesian Information Criterion (SABIC), entropy, class size, and average class probabilities. We used the following criteria to select the best model: (1) values closer to 0 for the AIC and SABIC, (2) entropy values >0.8, (3) on-diagonal class probabilities >0.7, and (4) the smallest class in the model being at least 5% of the total sample to avoid over-extraction. All eight HRSN items were used in addition to demographic variables, and location of HRSN screening (i.e., inpatient vs. outpatient). All variables were included (e.g., sex assigned at birth, race, ethnicity, age, HRSN items) that would be used in all analyses to maximize the amount of data that could be used for imputing values. A logistic regression was utilized afterward to assess the ability of the model to differentiate between classes of individuals who were more at-risk as measured by SVI rank.

## Results

The overall sample included screening results from 302,929 adult patients. Patients were relatively evenly distributed across age range: 33% of included patients were between 19 and 44 years old, 32% were between 45 and 64 years (32%), and 30% were between 65 and 84 years old (30%), with the remaining 4% being 85 years or older. Most patients identified as female (59%), as White (66%), and as non-Hispanic (87%). Across healthcare settings, 81% were taken from outpatient services (*n* = 245,562) while approximately 19% were taken from inpatient settings (*n* = 57,367). Most patients were in the low (32%) and low-medium (27%) SVI areas with the remaining third in the medium high (18%) and high (15%) SVI areas. See Table 2 for complete demographic information.

**Table 2:** Patient demographics (*N* = 302,929).

Variable	*N*	%
Age Group		
19–44 years	100,314	33.1
45–64 years	97,387	32.1
65–84 years	91,181	30.1
85+ years	12,753	4.2
Missing	1,294	0.4
Race with Ethnicity		
White	198,949	65.7
Black or African American	59,905	19.8
Asian	17,154	5.7
American Indian or Alaska Native	468	0.2
Native Hawaiian or Other Pacific Islander	259	0.1
Two or More Races	2,972	1.0
Other/Unknown/Missing	23,222	7.7
Sex		
Female	179,118	59.1
Male	123,752	40.9
Missing	59	0.0
SVI		
1—Low (0.00–0.24)	97,385	32.1
2—Low Medium (0.25–0.49)	80,336	26.5
3—Medium High (0.50–0.74)	55,388	18.3
4—High (0.75–1.00)	45,548	15
Missing	24,272	8.0
Encounter Type		
Inpatient Visit	57,367	18.9
Outpatient Visit	245,562	81.1

Positivity ranged between 0.90%–5.90% (i.e., lonely, 5.9%; skip doctors' visits, 3.4%; food insecurity, 2.7%; skip medication, 2.1%; utilities, 2.0%; housing instability, 1.9%; transportation, 1.8%; safety, 0.9%). Missing data ranged between 2.90%–7.40% for each item. Inability to assess or patient decline ranged between 2.60%–3.70%. Only those patients with at least one positive screening response were asked follow-up questions about whether they needed help or the need was urgent. Of the 91,292 patients who responded to the question about needing help, 11,455 (12.5%) answered “yes.” Of the 76,251 who responded to the question about having urgent needs, 4,964 (6.5%) answered “yes.”

### Item response theory results

Table 3 provides information about factor loadings and 2PL model parameters. In Mplus, pooling is automatically calculated for factor loadings using Rubin's rules across all imputed datasets with some limitations. However, it cannot do this for both item difficulty and item discrimination. These variables were averaged across all five datasets. Each item had a factor loading between.68 and.90. The columns labeled “slope” and “difficulty” provide IRT estimates of discrimination and the point on the latent trait scale where the probability of responding is 50% (difficulty). The slope/discrimination parameters are all positive and large ranging between 1.67–3.77. This means that each item sharply discriminates between high and low levels of the latent variable, which is HRSN. The difficulty parameters are also all positive and large ranging between 2.20–3.31. The scale of the latent variable in IRT is arbitrary, usually set to have a mean of 0 (i.e., those with “average” need would have latent scores of 0, those with low need would have latent scores <0 and those with high need have scores >0).

**Table 3:** Factor loadings and item response theory (IRT) parameters (*N* = 302,929).

Item	Factor loading	*a*	*b*
Multiple Imputation across all 10 datasets			
Do You Skip Medication to Save Money?	0.81	2.54	2.47
Do You Skip Doctors Visits to Save Money?	0.82	2.62	2.18
Did You Ever Eat Less Than You Should Because There Wasn't Enough Money For Food?	0.90	3.77	2.13
Are You Worried That In The Next 2 Months You May Not Have Stable Housing?	0.87	3.12	2.38
In The Last 12 Months Have Utilities Been Threatened To Be Shut Off In Your Home?	0.80	2.39	2.56
In The Last 12 Months Have You Ever Had To Go Without Healthcare Because You Didn't Have A Way To Get There?	0.84	2.77	2.49
Are You Concerned About Your Safety In Your Home?	0.72	1.89	3.31
Do You Often Feel Lonely?	0.68	1.67	2.20

IRT, parameters.

*a,* slope/discrimination; *b*, item difficulty.

### Latent class analysis results

Four LCAs were conducted across the 5 generated datasets. When using an imputation command, Mplus pools all data automatically to produce AIC, SABIC, final class counts and proportions, entropy, estimates, and probabilities. Thus, it generates a singular output for each analysis. Per best practices, latent class analyses were iteratively conducted across three models: 2-class, 3-class, and 4-class solutions. See Table 4 for the final LCA results pooled across all datasets. The 2-class model fit the data best (i.e., entropy >0.80 and on-diagonal class probabilities >0.70). The 3-class and 4-class models had entropy values below 0.80 and the AIC and SABIC values were comparable to the 2-class model, indicating that these models did not improve fit. Class 1, High Need, was characterized by being the smallest class (*n* = 17,049, 6%). When compared to Class 2, this class had a higher probability of being female (0.66), being Black (0.42), being Hispanic (0.17), and being between the ages of 18 and 44 (0.52). In terms of HRSN items, this class had the highest probabilities of having individuals endorse each item on the measure (e.g., skipping doctors' visits, eating less because there wasn't enough money, being worried about not having stable housing). Class 2, Low Need, was characterized by being the largest class (*n* = 285,880, 94%). This class had the highest probability of being male (0.41), being White (0.72), and not identifying as Hispanic (0.92). Notably, Class 2 had similar probabilities for individuals across most age groups (i.e., 19–44, .319; 45–64, .320; 65–84, .315) except for individuals 85+ (0.045). In terms of HRSN items, this class had the lowest probabilities of having individuals endorse each item on the measure.

**Table 4:** Results from the latent class analysis with two classes present.

2-Class solution	Class 1	Class 2
Sex		
Female	**.658**	.587
Male	.342	**.413**
Race		
White	.501	**.720**
Black	**.418**	.203
Asian	.044	**.065**
American Indian	**.005**	.002
Native Hawaiian/Pacific Islander	**.002**	.001
Two or More Races	**.029**	.010
Ethnicity		
Hispanic/Latino	.828	**.924**
Not Hispanic/Latino	**.172**	.076
Age		
19–44	**.519**	.319
45–64	**.359**	.320
65–84	.118	**.315**
85+	.005	**.045**
Type		
Inpatient	.139	**.193**
Outpatient	.259	**.807**
HRSN		
Skip Medications	**.259**	.006
Skip Doctors' Visits	**.417**	.009
Food Insecurity	**.381**	.003
Unstable Housing	**.264**	.003
Utilities Being Shut Off	**.255**	.005
Go Without Healthcare	**.228**	.004
Safety Concerns	**.097**	.003
Lonely	**.431**	.040

Bolded values indicate the highest probabilities.

## Discussion

In this study, we establish validity of the Jefferson HRSN screener for administration in both inpatient and outpatient healthcare settings to identify patients with HRSN. Findings suggest that the eight question screener has construct validity and preliminary evidence for predictive validity in detecting high levels of HRSN. The steep slopes (1.67–3.77) found in the IRT analysis indicated that the measure detects individuals who had heightened HRSN. Additionally, no slopes were greater than 4, indicating that that items did not covary. Specifically, items related to food and housing insecurity may be more discriminatory than questions related to loneliness and safety (31). The LCA and logistic regression analyses demonstrated that the measure could be used to help classify and identify people who were more at-risk for HRSN. The 2-class solution identified two latent classes with different response patterns, a larger class with low probabilities of having HRSN and a smaller class with high probabilities of having HRSN. Additionally, this analysis found that the two classes differed in terms of SVI rank areas.

Our findings add to the literature by providing a validated screening tool for assessing HRSN in both inpatient and outpatient healthcare settings immediately available for uptake by other health systems. This tool was developed by our health system leadership and was refined based on end user clinician and patient feedback, with the goal of having a screener that is usable, acceptable and useful as a routine measure in both inpatient and outpatient settings. Moreover, questions were designed to be understandable and actionable. This is the first study to our knowledge that has employed advanced statistical methods to validate the measure. This psychometric analysis in combination with our careful attention to clinic workflow concerns and clinician and patient feedback makes this screener a robust option for health systems. Validation of this HRSN screener is timely with recent CMS requirements that health systems report on screening for social drivers of health during acute care hospital admissions. Additionally, primary care payment models offer new supports to screen for and address HRSN during outpatient visits with a validated screening tool.

There are very few validated HRSN screeners to date, and none of which we are aware validated for use across both inpatient and outpatient healthcare settings; this work adds an additional validated screening tool—the Jefferson HRSN screener—to the literature, using robust measures of assessment, making it freely available for uptake by other health systems. Routine use of this screener in healthcare encounters can provide valuable information at both the individual and broader system level. Specifically, our results indicate that the screener could be used to identify populations that are more at-risk for HRSN. Our model showed that screening positive at the item level, identifying as a racial or ethnic minority, being female, and being younger were all associated with higher SVI rank. Patient-level information provides clinical teams with additional considerations as we work to improve outcomes. System-level information on predominant HRSN needs provides important insights on higher level tactics and programs that should be prioritized to address specific community needs.

This work has several limitations. Prior literature has documented multiple limitations to conducting social needs screening, including stigma that limit patient comfort in reporting existing need and lack of sufficient staff time and buy-in for conducting screenings (32–34). While expectations for routine screening with this Jefferson HRSN screener were uniformly established across the Jefferson enterprise, it is possible that various factors could contribute to patient needs being underreported in the data used. Efforts to address this in our setting include options for patient self-completion of the screener in the outpatient setting ahead of their visit or during their own check-in process and reducing other nursing documentation in the acute care environment when the HRSN screener was added to the admission workflow. Additionally, this analysis was done after the health system had launched an aligned HRSN screening approach in spring 2023 and was not conducted as a prospective research study. Therefore, measures available for validation were limited to those available using data in the medical record. In addition, details of the patient feedback and other factors informing modification of the screener questions are limited because the screener was refined by leadership in response to real time clinical needs as opposed to as part of a rigorous research study. Lastly, as the goal of this work was to assess validity across a large economically and racially diverse population, assessing specific responses across multiple subgroups (e.g., Medicare beneficiaries) was beyond the scope of this manuscript. For instance, this analysis indicated that 2% of individuals skip medications to save money while other analyses have found that rates of skipping medications may occur between 14%–30% in various subgroups (e.g., Medicare beneficiaries). Despite these limitations, this work provides sufficient evidence of validity for the Jefferson HRSN screener to support its continued use as a routine screener to identify HRSN for those seeking acute support in the healthcare setting.

In conclusion, the Jefferson HRSN screener, taken in concert with evaluating the patient's desire for help and urgency, can be used to help triage patients within healthcare settings seeking support to appropriate resources. This screener fills a critical gap in the literature at a time in which there is increasing national emphasis on the need for health systems to conduct routine HRSN screenings with validated instruments. Implementation of a tool such as the Jefferson HRSN screener is an important step in health systems' efforts to identify and address fundamental drivers of health disparities.

## Data Availability

The raw, cleaned data supporting the conclusions of this article will be made available by the authors, without undue reservation.
